# Field experience with two different vaccination strategies aiming to control infections with *Actinobacillus pleuropneumoniae *in a fattening pig herd

**DOI:** 10.1186/1751-0147-52-23

**Published:** 2010-03-25

**Authors:** Marie Sjölund, Per Wallgren

**Affiliations:** 1 National Veterinary Institute, Department of Animal Health and Antimicrobial Strategies, SE-751 89 Uppsala, Sweden; 2 Swedish University of Agricultural Sciences, Department of Clinical Sciences, PO Box 7054, SE-750 07 Uppsala, Sweden

## Abstract

**Background:**

The prevalence of pleurisies recorded at slaughter is increasing in Sweden, and acute outbreaks of actinobacillosis that require antimicrobial treatments have become more frequent. As an increased use of antimicrobials may result in the development of antimicrobial resistance it is essential to develop alternative measures to control the disease. Vaccinations present an appealing alternative to antimicrobial treatments. The aim of this work was to evaluate the potential of two different vaccination strategies in a specialized fattening herd affected by actinobacillosis.

**Methods:**

The study was conducted in a specialized fattening herd employing age segregated rearing in eight units. The herd suffered from infections caused by *Actinobacillus pleuropneumoniae *serotype 2, confirmed by necropsy and serology. The study included 54 batches of pigs grouped into five periods. Batches of pigs of the second period were vaccinated against actinobacillosis twice, and pigs in the fourth period were vaccinated three times. Batches of pigs of the first, third and fifth period were not vaccinated. Concentrations of serum antibodies to *A. pleuropneumoniae *and serum amyloid A (SAA) were analysed and production data were recorded.

**Results:**

Despite vaccinating, medical treatments were required to reduce the impact of the disease. The mean incidence of individual treatments for respiratory diseases during the rearing period ranged from 0 to 4.7 ± 1.8%, and was greatest during the triple vaccination period (period IV; p < 0.05 when compared to other groups). A large proportion of the vaccinated pigs seroconverted to *A. pleuropneumoniae *serotype 2 in the absence of a SAA-response. The prevalence of pleuritis decreased from 25.4 ± 6.5% in the first period to 5.0 ± 3.7% in the fifth period (p < 0.001).

**Conclusions:**

The vaccine did not effectively prevent clinical expression of *A. pleuropneumoniae *infections, but seroconversion to *A. pleuropneumoniae *in the absence of a SAA-response in a large number pigs indicated that the vaccine had activated the immune system. Further, the prevalence of pleuritis decreased with time. This indicates that vaccinations together with intensified medical treatments of affected pigs could be useful in reducing the impact of *A. pleuropneumoniae *serotype 2 infections.

## Background

*Actinobacillus pleuropneumoniae *is a causative agent of respiratory disease in pigs with symptoms ranging from sudden deaths to subclinical disease detected as pleurisies in the post mortem inspection at slaughter [1]. Infections with *A. pleuropneumoniae *may cause great economic losses due to mortality, increased feed consumption, retarded growth rate and medication [1-3]. Several strategies have therefore been employed aiming to control the effects of *A. pleuropneumoniae *infections of which age segregated rearing is one [4,5]. The ban on the use of growth promoters in Sweden in 1986 led to a more consistent implementation of age segregated rearing systems [6] which reduced the incidence of pleurisies recorded at slaughter from 8% in 1988 to 5% in 2002 [7]. However, registrations for pleurisies at slaughter are currently increasing and acute outbreaks of actinobacillosis are becoming more frequent [8]. Such outbreaks often require antibiotic treatment of entire units with in-feed medication which has been mirrored by an increased prescription of tetracyclines in 2007 [9].

To date, none of the tested Swedish isolates of *A. pleuropneumoniae *have been resistant to the antibiotics tested for [9]. Despite this, it is essential to develop antibiotic independent measures to control the disease since an increased use of antibiotics may promote the emergence of antimicrobial resistance [10]. Antimicrobial resistance for *A. pleuropneumoniae *isolates has been reported [11].

Vaccination presents an appealing alternative to antibiotics in reducing the impact of *A. pleuropneumoniae*. The first generation of vaccines against *A. pleuropneumoniae *did not provide sufficient protection against disease and were in some cases causing adverse side effects such as depression, inappetence, fever or tissue damage [12]. At present, one subunit vaccine is commercially available in Sweden (Porcilis^® ^APP, Intervet, Boxmeer, The Netherlands). Several reports from different countries have described the efficacy of this vaccine [13,14]. According to the product details, this vaccine induces a gradually developing protective immunity which is greatest two to three weeks after booster vaccination with some protection maintained for up to seven weeks. This work aimed at evaluating the effect over time of two different vaccination strategies in a specialized fattening herd affected by actinobacillosis.

## Methods

### Herd and batches followed in a longitudinal survey

The study was approved by the Ethical Committee on Animal Experiments, Uppsala, Sweden (Licence C38/4). It was conducted in a conventional, specialized fattening herd producing approximately 7500 pigs per year. The herd was free from all diseases listed by the Office International des Epizooties, Paris, France, and also from Aujeszky's disease, PRRS and *Salmonella*. However, the herd had suffered from infections caused by *Actinobacillus pleuropneumoniae *serotype 2 for two years, which had been confirmed by necropsy and serology. Batch prevalence at slaughter for pleurisy lesions ranged from 18.7% to 26.8% and for *Mycoplasma hyopneumoniae*-like lesions from 1.7% to 19.2% during the years preceding the study (see also Table 1).

**Table 1:** Lesions of the respiratory tract registered at slaughter in fatteners unvaccinated or vaccinated against *Actinobacillus pleuropneumoniae *in a specialized fattening herd affected by actinobacillosis

Batch Category	# batches	Mycoplasma-like pneumonia	Pleuritis	Hemmorrhagic broncho-pneumonia
		(%)	(%)	(%)
Period I Before vaccinations	6	7.9 ± 8.1	25.4 ± 6.5	0 ± 0
Period II Double vaccinations	13	10.7 ± 5.6	19.7 ± 8.1	2.5 ± 5.0
Period III In between vaccinations	11	14.8 ± 9.6	13.9 ± 3.9	0.5 ± 0.9
Period IV Triple vaccinations	8	7.4 ± 2.3	11.1 ± 2.8	1.0 ± 1.1
Period V After vaccinations	16	9.7 ± 4.3	5.0 ± 3.7	0.6 ± 1.0

**Figure 1:**
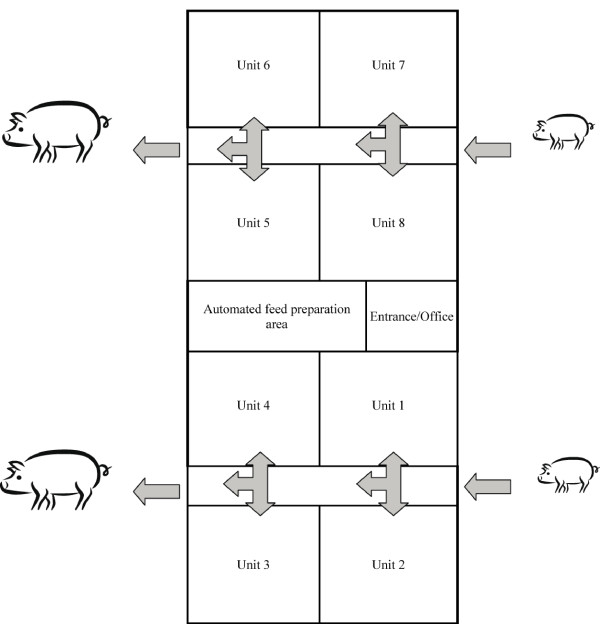
**A schematic view of a specialized fattening herd employing strict all in - all out production with a turn-over time of 16 weeks per unit**. Each of the eight units housed 11 pigs per pen in 32 pens (n = 352 pigs per unit).

Pigs were housed in a 10 year old building with eight units (Figure 1). Each unit housed 11 pigs per pen in 32 pens (n = 352). Strict all in - all out production with a cycle of 16 weeks was employed in all units. Thus, a new batch of pigs entered a thoroughly cleaned and disinfected unit every second week. The pigs arrived at an age of 10-12 weeks with two or three suppliers per batch. Antimicrobial substances were not routinely added to the feed given to the pigs.

This study included 54 batches of pigs that were grouped into five successive periods. The first period (I) included six unvaccinated batches. The second period (II) included 13 batches that were vaccinated twice against actinobacillosis (see below). The third period (III) included 11 unvaccinated batches. The fourth period (IV) included eight batches vaccinated three times against actinobacillosis. The fifth period (V) included 16 unvaccinated batches.

### Vaccine and vaccination strategies employed

A commercially available vaccine (Porcilis^® ^APP Intervet, Boxmeer, The Netherlands), containing three inactivated exotoxins (ApxI, ApxII, ApxIII) and a 42 kDa outer membrane protein (OMP) was used employing two different strategies.

Thirteen consecutive batches (period II) were vaccinated twice with 2 ml of Porcilis^® ^APP. The first two vaccinated batches were already present on the premises when the vaccination scheme was initiated. Thus, Batch 1 and Batch 2 of period II received the first vaccination 20 and 35 days after arrival, respectively. The other 11 batches were vaccinated on arrival. Booster vaccinations were performed 28 days after the first vaccination. Employing a second strategy (period IV), pigs were vaccinated three times with 2 ml of Porcilis^® ^APP during one turn-over of the herd, *i.e*. until pigs in all eight units had been vaccinated. The first vaccination was given on arrival to the fattening herd. Vaccinations were repeated 28 and 56 days after arrival.

### Blood sampling procedures

Blood samples without additives were collected from pigs by jugular vein punctures using evacuated plastic tubes (BD Vacutainer Systems, Belliver Industrial Estate, Plymouth, United Kingdom). They were centrifuged for 10 minutes at 800 × g, after which the serum was removed and stored at -20°C until analysed.

A cross-sectional blood sampling comprising six pigs per age category/unit was performed before initiating the vaccination strategies in order to obtain a serological profile of the herd. This sampling was performed in connection with an outbreak of acute pleuropneumonia.

The blood sampling procedure for the four batches of vaccinated pigs during period II and period IV were identical except for day 56. Blood samples were repeatedly collected from individually ear-tagged pigs every fortnight. Blood samples collected on days 0, 28 and 56 were collected before pigs were vaccinated. Blood was collected from pigs in the eighth and tenth batches of pigs vaccinated twice (Period II: Batch 8; n = 31 and Batch 10; n = 15). From pigs vaccinated three times, blood was collected from the first and last batch (Period IV: Batch 1, n = 30; Batch 8, n = 21).

### Detection of antibodies to A. pleuropneumoniae serotype 2

An indirect ELISA, based on a phenol-water extraction of the microbe as coating antigen, was used to measure serum antibodies to *A. pleuropneumoniae *serotype 2 in all serum samples collected. The cut-off value for a positive reaction in sera diluted 1/1000 was defined as A_450 _= 0.5 [15].

### Serum Amyloid-A (SAA)

Serum levels of the acute phase protein Serum Amyloid-A (SAA) were analysed using a commercial kit (Serum Amyloid A Assay TP-802, Tridelta, Maynooth, Ireland) according to the instructions of the producer. The results are presented as mg SAA per ml serum. The baseline serum levels of SAA were established using sera from 30 nine-week-old specific pathogen-free (SPF) pigs [16]. The mean SAA serum levels of these pigs were 37.8 mg per ml (Max - min range: 22.7 - 157.2 mg per ml), and considered as representative serum levels of SAA for healthy pigs. As cut-off for an increased level of SAA in the present study the 95% percentile (70 mg per ml) of the serum level of SAA of the SPF pigs was used.

SAA was analysed in the serum samples collected from six pigs per age category in the cross-sectional sampling performed before the period of investigation. SAA was also analysed in all serum samples collected from ten selected pigs per batch of the four sampled vaccinated batches of period II and IV.

### Clinical recordings and medical treatments

The herd veterinarian made regular visits to the herd. Routine herd procedures included daily inspections with disease monitoring performed by the farm manager according to instructions from the herd veterinarian. During the period of investigation, all pigs present on the premises were inspected by the investigating veterinarian on blood sampling occasions. Individual pigs with signs of respiratory disease were treated intramuscularly with oxytetracycline for five days (20 mg/kg body weight once daily on days 1, 3 and 5; Engemycin^® ^vet., Intervet, Boxmeer, The Netherlands) according to written instructions from the herd veterinarian. In severe cases with many pigs affected by respiratory disease and per-acute mortalities, affected batches were in-feed medicated with either 20 mg chlortetracycline per kg body weight and day (Clortetraciclina 20%, Ceva Sante Animale, Libourne Cedex, France) or 12.5 mg doxycycline per kg body weight and day (Pulmodox 5%, ChemVet, Silkeborg, Denmark) for 10 consecutive days. In-feed medications were initiated and prescribed by the herd veterinarian.

### Registrations at slaughter and production data

The incidence of lung lesions (enzootic pneumonia, pleuritis and necrotizing bronchopneumonia) was obtained from the regular meat inspection performed at the abattoir. Data of average daily weight gain (DWG) was obtained from the production control system used by the herd (FarmData, BioManagement AB, Tumba, Sweden). These data were collected from all 54 batches include in the study (period I to V).

### Statistical analysis

All results in the text are given as mean values ± standard deviations. Continuous non-normally distributed data was analyzed using the Wilcoxon Rank Sum test and non-continuous data were categorized and analyzed using the Fishers Exact test.

## Results

### Serology

**Figure 2:**
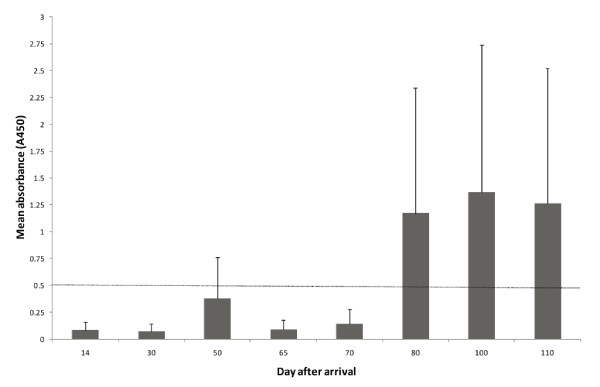
**A cross-sectional serological screening for mean serum antibody levels (A_450_) to *Actinobacillus pleuropneumoniae *serotype 2 performed during an outbreak of actinobacillosis in a specialized fattening herd employing age segregated rearing**. Six pigs per unit were analysed. The error bars show positive standard deviations and the dotted line indicates the cut-off value for a positive reaction.

Pigs that had been at the herd for less than 80 days were in general serologically negative to *A. pleuropneumoniae *serotype 2 in the cross-sectional sampling performed before commencing the study. However, some of the pigs that had been at the herd for 50 days were seropositive to *A. pleuropneumoniae *serotype 2. All but one of the pigs that had been at the herd for 80 days or more were serologically positive to *A. pleuropneumoniae *(Figure 2).

**Figure 3:**
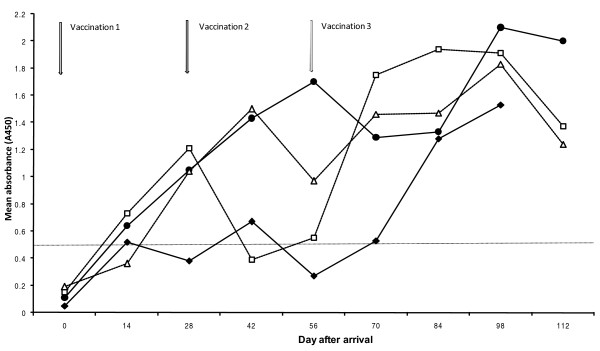
**Mean serum antibody levels (A_450_) to *Actinobacillus pleuropneumoniae *serotype 2 in batches of pigs vaccinated two or three times against *Actinobacillus pleuropneumoniae *in a specialized fattening herd affected by the infection**. The first vaccination was performed as the pigs arrived at the specialized fattening herd. The booster vaccination(s) were carried out after intervals of 28 days, Double vaccinated pigs are shown by filled symbols ([black diamond] = batch 8 and [black circle] = batch 10. Pigs vaccinated three times are shown by open symbols (black triangle = batch 1 and black sqaure = batch 8). The dotted line indicates the cut-off value for a positive reaction.

With respect to pigs vaccinated twice (period II), all animals sampled were seronegative (A_450 _< 0.5) to *A. pleuropneumoniae *serotype 2 on arrival to the fattening herd. Following the initial vaccination, an increase (p < 0.001) in serum antibodies to *A. pleuropneumoniae *serotype 2 was observed after 14 days in both batches that were analysed. By this time, 11 out of 31 and 6 out of 15 pigs had seroconverted in Batch 8 and 10, respectively (mean A_450 _for seropositive pigs = 0.71 ± 0.29 in Batch 8 and 1.06 ± 0.33 in Batch 10). The mean absorbance value remained at that level until 84 days after arrival in Batch 8. In contrast, the mean absorbance value increased continuously to A450 = 1.70 ± 0.29 at day 56 in Batch 10 (Figure 3). At the last sampling occasion, all pigs but two pigs in batch 8 were seropositive to *A. pleuropneumoniae *serotype 2. The mean absorbance value for these two pigs was A_450 _= 0.31 ± 0.24.

The mean absorbance values for antibodies to *A. pleuropneumoniae *serotype 2 in the first and last batches vaccinated three times (period IV) are also shown in figure 3. In total, two out of 30 pigs in batch 1 and four out of 21 pigs in batch 8 were seropositive to *A. pleuropneumoniae *serotype 2 on arrival to the fattening herd with a mean absorbance level of A_450 _= 0.76 ± 0.29. The mean absorbance value increased significantly (p < 0.05) between sampling times until day 28 in both batches, and Batch 1 continued to increase until day 42. Following the third vaccination (day 56), the amount of serum antibodies to *A. pleuropneumoniae *serotype 2 had increased (p < 0.01) at the next sampling for both batches (day 72). A decrease in the level of serum antibodies to *A. pleuropneumoniae *serotype 2 was observed at the last sampling occasion (day 112) compared to the previous sampling occasion (day 98: Figure 3). This decrease was significant for Batch 1 (p < 0.001). However, at this occasion all but one pig (A_450 _= 0.24) were seropositive to *A. pleuropneumoniae *serotype 2.

### SAA and the relation to seroconversion to A. pleuropneumoniae

In total, increased serum levels of SAA were recorded in seven out of the 48 pigs in the cross-sectional sampling. Two of these pigs had been in the herd for 80 and 100 days, respectively. The remaining five pigs with elevated serum levels of SAA (mean = 969 ± 618 mg per ml) had been in the herd for 50 days. At this time point, three of these pigs had seroconverted to *A. pleuropneumoniae *and the mean absorbance level for antibodies to *A. pleuropneumoniae *was 0.38 ± 0.34 for these six pigs (Figure 2). In contrast, pigs that had been at the herd for 30 days had lower serum antibody levels to *A. pleuropneumoniae *(mean A_450 _= 0.07 ± 0.02) and none of these pigs had elevated serum levels of SAA.

Elevated serum concentrations of SAA were detected in individual vaccinated pigs throughout the rearing period. Increased serum levels of SAA were demonstrated in 16 out of 20 pigs vaccinated twice (period II), and on an individual basis, elevated SAA concentrations were recorded on one to seven occasions. In pigs vaccinated three times (period IV) increased serum levels of SAA were demonstrated in 14 out of 20 pigs (one to three occasions per pig).

Seroconversion to *A. pleuropneumoniae *serotype 2 was observed at the sampling occasion following the sampling when elevated SAA concentrations were observed in eight of 20 pigs that had been vaccinated twice (40%) and in six of 20 pigs that had been vaccinated three times (30%). Thus 12 of 20 pigs vaccinated twice (60%) and 14 of 20 pigs vaccinated three times (70%) seroconverted to *A. pleuropneumoniae *serotype 2 in the absence of a SAA-response at the previous sampling occasion (Table 2).

**Table 2:** Elevated serum levels of SAA related to seroconversion to *Actinobacillus pleuropneumoniae *serotype 2 in pigs vaccinated either two or three times against actinobacillosis

Elevated levels of SAA	Period II Vaccinated 2 times	Period IV Vaccinated 3 times
	(number of pigs = 20)	(number of pigs = 20)
Not recorded	4 (20%)	6 (30%)
4 - 8 weeks prior to seroconversion	2 (10%)	4 (20%)
2 weeks prior to seroconversion	8 (40%)	6 (30%)
Simultaneous to seroconversion	6 (30%)	0
After seroconversion	0	4 (20%)

### Clinical recordings and treatments

Historically, clinical symptoms of respiratory disease had rarely been observed, and medical treatments against respiratory diseases were generally not carried out (Table 3). However, due to clinical disease, in-feed medications with doxycycline were required. Five consecutive batches of pigs were medicated: three batches in period I and the first two batches of pigs vaccinated twice (period II). In period II, 1.3 ± 2.0% of the pigs also required individual treatments due to respiratory disease. The mean mortality during this period when pigs were vaccinated twice amounted to 4.0 ± 1.3% (Table 3).

**Table 3:** Origin of growers, daily weight gain, medical treatments against respiratory disease and mortality in fatteners unvaccinated or vaccinated against *Actinobacillus pleuropneumoniae *in a specialized fattening herd affected by actinobacillosis

Batch Category	# batches	Origin of growers	Weight gain	Treatments Individual	Treatments In feed	Mortality
		(Herds)	(g per day)	(%)	(Batches)	(%)
Period I Before vaccinations	6	A, B, C	863 ± 56	0 ± 0	3 of 6	4.2 ± 1.6
Period II Double vaccinations	13	A, B, C	911 ± 34	1.3 ± 2.0	2 of 13	4.0 ± 1.3
Period III In between vaccinations	11	A-F	887 ± 16	1.3 ± 2.0	0	3.7 ± 1.7
Period IV Triple vaccinations	8	D, E, F	893 ± 33	4.7 ± 1.8	6 of 8	3.1 ± 1.4
Period V After vaccinations	16	D, E, F	883 ± 36	0.6 ± 0.9	0	3.4 ± 1.3

Individual treatments for respiratory symptoms ranged from zero to 23 pigs per batch (0 - 6.7%) for the 11 batches with unvaccinated pigs during period III (mean = 1.3 ± 2.0%). The mean mortality for these batches was 3.7 ± 1.7%.

The incidence of individual treatments for respiratory diseases was greatest during the triple vaccination period (period IV), (p < 0.05 when compared to period I, II, III and V). Four to 22 pigs per batch were individually treated for respiratory disease symptoms (mean = 4.7 ± 1.8%). The first individual treatments were initiated 18 to 24 days after arrival to the herd. Due to the large number of individual treatments for respiratory disease and per acute mortalities, in-feed treatment with chlortetracycline was applied at the onset of clinical signs, approximately three weeks after arrival in all but two of the eight batches. The overall mortality for the eight batches of pigs vaccinated three times was 3.1 ± 1.4%.

After the cessation of vaccinations (period V), zero to nine pigs per batch in 16 consecutive batches received individual treatments for respiratory disease (mean = 0.6 ± 0.9% treatments. The mean mortality for these batches was 3.4 ± 1.3% (Table 3).

### Registrations at slaughter and growth performance

The registrations at slaughter of the pigs are shown in Table 3. Registrations for Mycoplasma-like pneumonias and for hemorrhagic bronchopneumonias varied over time. In contrast, the prevalence of pleuritis was lower (p < 0.05) for the unvaccinated pigs of period III compared to pigs vaccinated twice (period II). The prevalence of pleuritis registered at slaughter was lower in period V compared to all other periods (p < 0.01).

The daily growth during the rearing period ranged from 863 ± 56 to 911 ± 34 g per day. The differences in growth rate observed were not statistically significant when the periods were compared to each other (Table 1).

## Discussion

This work was initiated due to an outbreak of acute actinobacillosis confirmed through necropsy and serological screening in a fattening herd suffering from chronic pleuropneumoniae. However, despite double vaccinations (period II), medical treatments were required to reduce the impact of the disease. As the protection of the vaccine has been reported to be of rather short duration and greatest two to three weeks after a booster vaccination [17], it was assumed that three vaccinations would prolong the period of protection. Yet, individual treatments of pigs for respiratory disease and in-feed medications were required in six of the eight batches that were vaccinated three times (period IV). On the other hand, the pleurisy registrations at slaughter decreased significantly in batches following vaccinated ones. Similar results have also been reported by others [18], which could be an indication of a reduction in the pathogen load, not apparent until after terminating the vaccinations. However, these authors did not observe any differences in growth performance and pleurisy lesions recorded at slaughter between vaccinated and control pigs. Most likely the individual and the in-feed medications contributed to the reduced pathogen load, and it appears that vaccinations together with intensified medical treatments of affected pigs could be useful in reducing the impact of *A. pleuropneumoniae *infections as also previously suggested [19].

The serological results from the batches vaccinated twice (period II) indicated that transmission not only occurred between pigs, but also between units. Airborne transmission of *A. pleuropneumoniae *between closely located units has been reported to occur under experimental conditions [20,21] and *A. pleuropneumoniae *is readily transmitted between pigs [20,22]. *A. pleuropneumoniae *can also be transmitted between units in large herds housing several age categories in the same building even when all-in all-out management is effectuated on room basis [23]. Indeed, a higher proportion of batches were infected with *A. pleuropneumoniae *in systems employing all in-all out on room basis compared to when all in-all out rearing was carried out by site [5]. Pigs in facilities housing several age categories, as in the herd investigated, will thereby risk to be repeatedly exposed to the microbe.

Nevertheless, pig to pig transmission should not be neglected. It has previously been demonstrated that *A. pleuropneumoniae *is most readily isolated in pigs aged 11 to 12 weeks [24], which coincides with the mixing of pigs from different sources on arrival to fattening herds. At this age, serum neutralizing antibody titres are generally low why pigs may be susceptible to infections [25]. Pigs that are seropositive to *A. pleuropneumoniae *have obviously been infected and could be contagious. During period IV, the herd received pigs that were seropositive to *A. pleuropneumoniae*. Despite this, the prevalence of pleuritis decreased during period IV. Although the pigs originated from the same sources during period IV and V, the prevalence of pleuritis decreased even further during period V. It was therefore concluded that the transmission between units was significant for maintaining a high pathogen load in the herd.

Increased SAA-levels were demonstrated in pigs that had been at the herd for 50 days in the cross-sectional sampling. The mean absorbance value of serum antibodies to *A. pleuropneumoniae *for these pigs (A450 = 0.38 ± 0.34) indicated that the entire group was about to seroconvert. A primary exposure to *A. pleuropneumoniae *serotype 2 has previously been shown to induce a significant but transient SAA-response [26] with a duration of approximately seven to ten days [27,28]. In contrast, pigs do not mount SAA-responses when re-exposed to *A. pleuropneumoniae *serotype 2 [28]. The interval of two weeks between samplings may have concealed SAA-responses in individual pigs, but the seroconversion to *A. pleuropneumoniae *without a preceding SAA-response in less than 50% of the vaccinated pigs indicated that the vaccine had triggered the immune system. The reoccurring SAA-responses in individual pigs in period II and IV instead indicated that other pathogens than *A. pleuropneumoniae *induced the SAA-responses in the vaccinated pigs.

Despite stimulating the immune system, a local IgA response is not necessarily induced by vaccines [29]. This may explain the repeatedly occurring signs of clinical disease, since IgA appears to be important in a first line of defense against acute actinobacillosis [30]. If so, this highlights the importance of pathogen load reducing efforts [31,32] including treatments of diseased pigs [33,34] in controlling actinobacillosis. This is further supported by the fact that vaccinations have previously been shown not to influence whether pigs become infected and/or infectious [35]. The pigs in this study did not seroconvert merely due to the vaccinations (Batch 8, period II; Figure 3), which has also been observed by others [36]. As pigs with low antibody levels to the Apx toxins can be protected against disease [37], serum antibodies may not be essential in providing protection against *A. pleuropneumoniae *infections. On the other hand, specific serum IgG antibodies have been reported to be important in protection against pleuropneumonia [38], and the levels of toxin-neutralizing antibodies in serum have been shown to influence the susceptibility to *A. pleuropneumoniae *infections [39].

The pigs were vaccinated on arrival to the fattening herd at an age of 10-12 weeks, which may have been a suboptimal point of time as it appears to be important to perform the first vaccination prior to exposure to a pathogen load sufficient to cause clinical disease. Indeed, some pigs vaccinated three times were seropositive to *A. pleuropneumoniae *on arrival. Obviously these pigs had been exposed to *A. pleuropneumoniae *and the stress induced by transportation, co-mingling and changes in feed at a time when serum-antibody levels generally were low made a spread of infection feasible [1,25,40], which could have influenced the outcome of the vaccinations.

However, similar results to ours have been reported when three vaccinations were performed at six, 10 and 14 weeks of age [18]. These authors detected maternal antibodies in serum at the age of six weeks which may have interfered with the immune response following vaccination. Maternally derived IgG antibodies are reported to have a suppressing effect on the synthesis of immunoglobulins by suckling piglets [41], and maternal antibodies to *A. pleuropneumoniae *have been detected in serum up to an age of at least eight weeks [39,42,43]. Thus, the age of six weeks may not either be an optimal time for performing the first vaccination against *A. pleuropneumoniae*. Administering the first dose at a later time point appears to be beneficial provided that the pigs are uninfected.

On the other hand, the presence of maternal antibodies has been shown not to hinder the induction of a specific primary antibody response when administering a low-dose infection [43]. The time point for immunization would thus not be crucial. In an endemically infected herd, pigs could already at the age of 11 days have been exposed to *A. Pleuropneumoniae *[44]. Thus, a carrier state can occur as the piglets harbour the microbe in the tonsils. On the other hand, disease is rarely seen while the piglets are still under maternal antibody protection [37,39]. As maternal immunity wanes, carrier pigs can transmit the infection to non-immune pigs [44,45], a situation likely to occur as pigs from several sources enter a unit at a fattening herd [6,46].

Thus, the future demands on vaccines are high. Subunit vaccines appear to convey better cross-protection than bacterins [47]. Still, subunit vaccines only provide partial clinical protection [48]. The use of live attenuated vaccines might better mimic a natural course of infection, with a potential to provide protection against heterologous challenge [49]. Further, as an intradermal administration route can induce both mucosal and cell-mediated immune responses [50,51], this could be a way to enhance the response to vaccination.

Another way to reduce the impact of an *A. pleuropneumoniae *infection under field conditions could be to ensure a high level of maternal antibodies in piglets by vaccinating the sows [52]. Maternal antibodies combined with a low-dose infection has been shown to be superior in protecting pigs from a challenge infection in comparison to either maternal antibodies alone or a low-dose infection in the absence of maternal immunity [43,53]. In endemically infected herds, pigs are likely to encounter a low-dose infection through asymptomatic carrier pigs, but this can also be achieved through attenuated live vaccines [49]. Further, an intra-nasal administration may provide enhanced protection against disease as local immunity has been shown to be important, improving the clearance of bacteria from the respiratory tract [12].

## Conclusions

In conclusion, the time point for immunization with the vaccine used in this study appeared not to be crucial as an immune response was induced, but still pigs were not protected against disease. Thus other disease preventing measures and treatments were also concluded to be essential in controlling *A. pleuropneumoniae *infections.

## Competing interests

The authors declare that they have no competing interests.

## Authors' contributions

MS and PW initiated the study and deigned it. MS effectuated collection of the blood samples and data collection. MS was head writer of the manuscript with help from PW. Both authors have read and approve the final manuscript.
